# Roles of LncRNAs in Regulating Mitochondrial Dysfunction in Septic Cardiomyopathy

**DOI:** 10.3389/fimmu.2021.802085

**Published:** 2021-11-24

**Authors:** Shuang Liu, Wei Chong

**Affiliations:** Department of Emergency, The First Affiliated Hospital of China Medical University, Shenyang, China

**Keywords:** lncRNA, epigenetics, sepsis, septic cardiomyopathy, mitochondrial dysfunction

## Abstract

Sepsis is an abnormal systemic inflammatory response of the host immune system to infection and can lead to fatal multiorgan dysfunction syndrome. Epidemiological studies have shown that approximately 10-70% of sepsis cases can lead to septic cardiomyopathy. Since the pathogenesis of septic cardiomyopathy is not clear, it is difficult for medical doctors to treat the disease. Therefore, finding effective interventions to prevent and reduce myocardial damage in septic cardiomyopathy is clinically significant. Epigenetics is the study of stable genetic phenotype inheritance that does not involve changing gene sequences. Epigenetic inheritance is affected by both gene and environmental regulation. Epigenetic studies focus on the modification and influence of chromatin structure, mainly including chromatin remodelling, DNA methylation, histone modification and noncoding RNA (ncRNA)-related mechanisms. Recently, long ncRNA (lncRNA)-related mechanisms have been the focus of epigenetic studies. LncRNAs are expected to become important targets to prevent, diagnose and treat human diseases. As the energy metabolism centre of cells, mitochondria are important targets in septic cardiomyopathy. Intervention measures to prevent and treat mitochondrial damage are of great significance for improving the prognosis of septic cardiomyopathy. LncRNAs play important roles in life activities. Recently, studies have focused on the involvement of lncRNAs in regulating mitochondrial dysfunction. However, few studies have revealed the involvement of lncRNAs in regulating mitochondrial dysfunction in septic cardiomyopathy. In this article, we briefly review recent research in this area.

## Sepsis and Septic Cardiomyopathy

Sepsis is an abnormal systemic inflammatory response of the host immune system to infection and can lead to fatal multiorgan dysfunction syndrome (1, 2). In severe cases, sepsis is considered a cause of death (3). Millions of human beings suffer from sepsis every year, and more than one-quarter of them lose their lives (4). According to statistics, the hospitalization rate and mortality of patients with severe sepsis increase by 8.2% and 5.6%, respectively, every year (5). Parker et al. first proposed in a 1984 study that sepsis-induced cardiac dysfunction is reversible (6). Since then, research on septic cardiomyopathy has attracted increasing attention. Epidemiological studies have shown that 10-70% of sepsis cases can lead to septic cardiomyopathy (7, 8). The mortality of patients with septic cardiomyopathy is 70%-90%, which is 2-3-fold higher than that of patients with sepsis that does not affect the heart (9, 10). Currently, there is no formal definition of septic cardiomyopathy. It is generally recognized that septic cardiomyopathy is transient cardiac dysfunction caused by sepsis and that it manifests as heart enlargement, ventricular systolic dysfunction, hypoperfusion without ventricular systolic dysfunction, poor response to fluid resuscitation and catecholamines, and so on (11–14).

**Figure 1:**
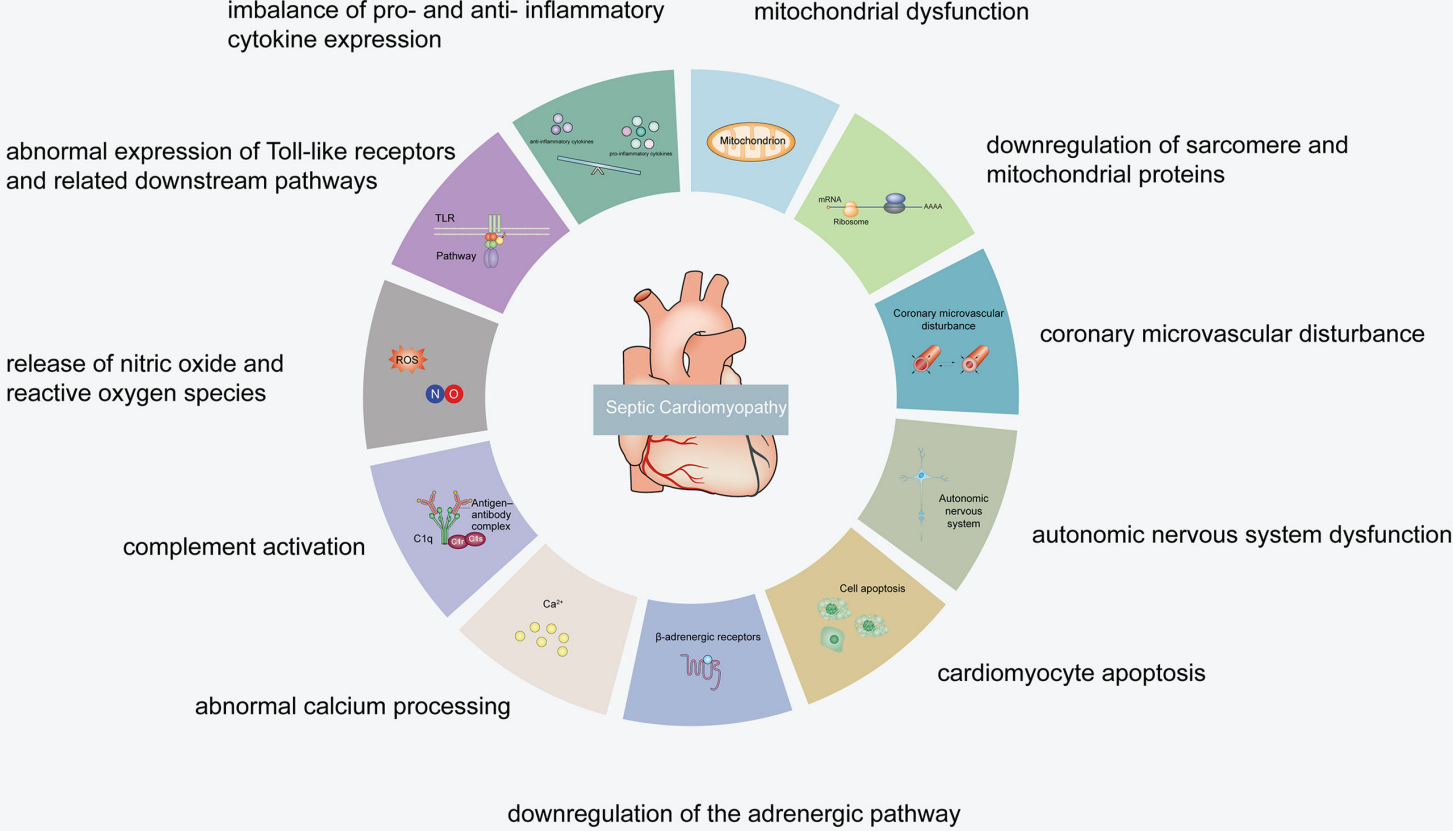
Septic cardiomyopathy pathogenesis.

**Figure 2:**
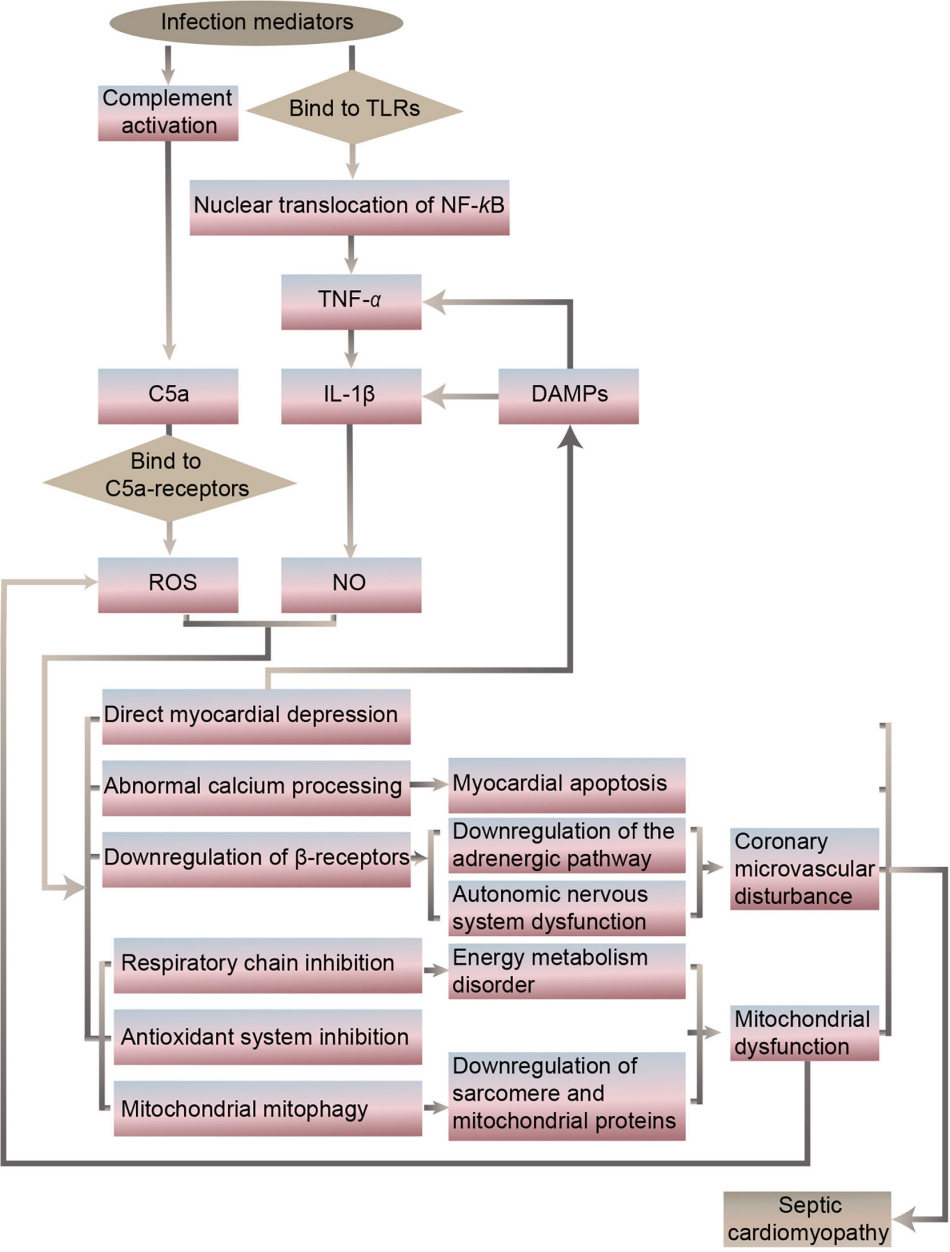
Potential targets of protective intervention in septic cardiomyopathy.

It has been revealed that the specific septic cardiomyopathy pathogenesis may include an imbalance of pro- and anti-inflammatory cytokine expression, abnormal expression of Toll-like receptors and related downstream pathways, release of nitric oxide (NO) and reactive oxygen species (ROS), complement activation, abnormal calcium processing, downregulation of the adrenergic pathway, cardiomyocyte apoptosis, autonomic nervous system dysfunction, coronary microvascular disturbance, mitochondrial dysfunction, and downregulation of sarcomere and mitochondrial proteins (15–18) (**Figures 1**, **2**).

## Mechanisms of Mitochondrial Dysfunction in Septic Cardiomyopathy

**Figure 3:**
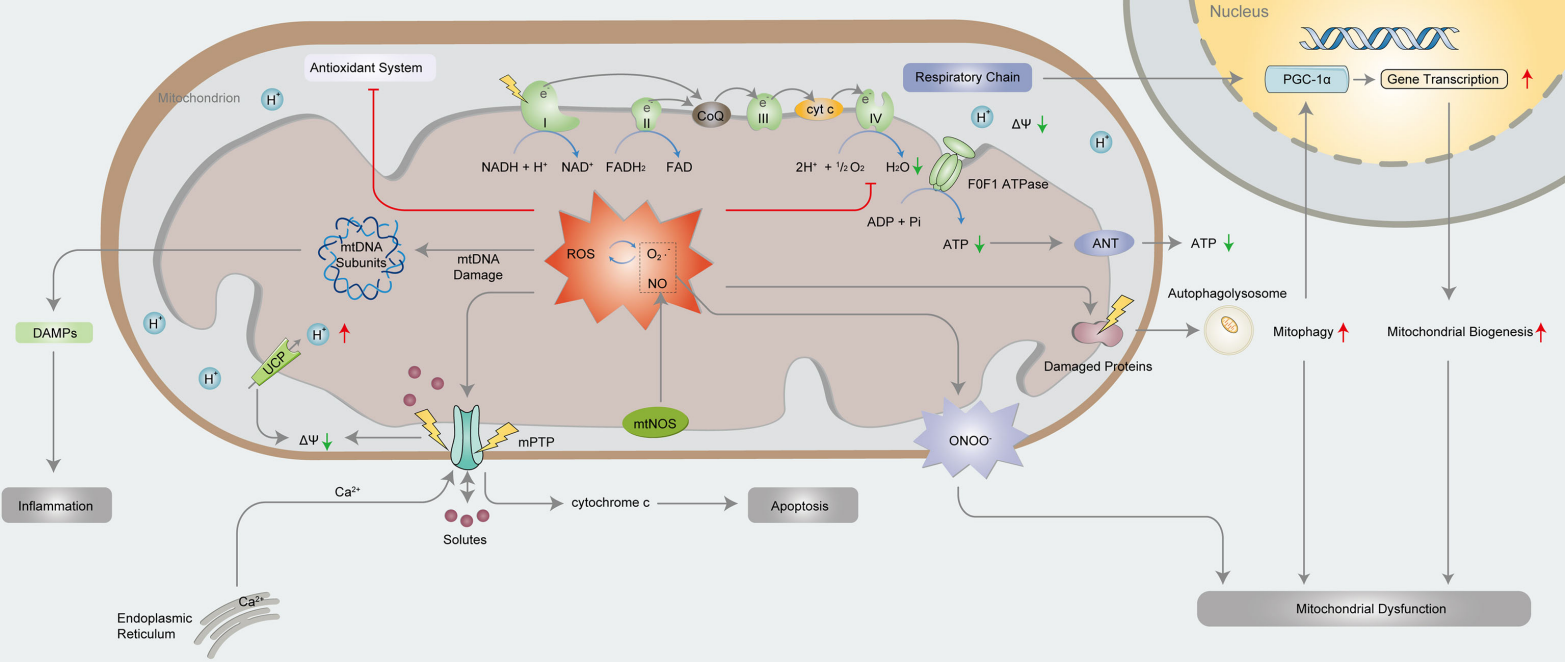
Mechanisms of mitochondrial dysfunction in septic cardiomyopathy.

Recently, researchers have focused on preventing and reducing myocardial damage in septic cardiomyopathy. Among the septic cardiomyopathy pathogenesis, mitochondrial dysfunction deserves to be a focus and further studied (19, 20) (**Figure 3**). Cardiomyocytes are rich in mitochondria, especially in the areas between sarcomeres and the subsarcolemma (21). As the energy metabolism centres of cells (22), mitochondria function to generate energy through oxidative phosphorylation (OXPHOS) (23). Of the important mechanisms of septic cardiomyopathy, the specific mechanism of mitochondrial dysfunction is under debate. Studies have shown that in the pathogenesis of septic cardiomyopathy, mitochondria undergo relevant changes that lead not only to mitochondrial dysfunction but also to the mitochondrial adaptive response (24, 25).

## Mitochondrial Ultrastructural Damage and Decreased ATP Production

In 1994, morphological damage of myocardial mitochondria in septic cardiomyopathy was first described in an animal model (26). Studies have shown that the ultra-microstructural abnormalities of myocardial mitochondria in septic cardiomyopathy include swelling, ridge loss, matrix clearance, rupture of internal vesicles, and damage to internal and external membranes (27, 28), which are closely related to mitochondrial dysfunction (29). Specifically, ultra-microstructural abnormalities lead to the destruction of the OXPHOS process and further reduce adenosine triphosphate (ATP) production. Mitochondria are critical for synthesizing more than 90% of the ATP required by the body (30). The role of the respiratory chain represents the basic function of mitochondria. The respiratory chain is mainly composed of complexes I, II, III and IV (31), and F0F1 ATPase (32). Fatty acid β-oxidation supplies nicotinamide adenine dinucleotide (NADH) and flavin adenine dinucleotide (FADH_2_), which respectively transport electrons for OXPHOS through complexes I and II. Subsequently, electrons are transported to complex III, and then to complex IV, leading to the reduction of O_2_ to H_2_O. Finally, ATP is generated under the effection of F0F1 ATPase in the mitochondrial inner membrane (33–35).

## NO Production and Oxidative Stress

Sepsis is accompanied by the excessive production of NO, ROS and inflammatory cytokines (36), leading to mitochondrial dysfunction (37). Mitochondria produce NO through mitochondria NOS (mtNOS), which inhibits cytochrome c oxidase to regulate mitochondrial respiration (38). NO and O_2_·^−^ produce ONOO^−^ through diffusion-controlled reactions (39). ONOO^−^ is a strong oxidant that can lead to direct oxidation or nitrosation damage, inhibit the OXPHOS complex and reduce O_2_ consumption (40, 41). Studies have shown that knockout of inducible NOS (iNOS) can attenuate injury induced by oxidative stress, impaired OXPHOS or reduced ATP synthesis, revealing the vital role of ONOO^−^ in regulating mitochondrial dysfunction in septic cardiomyopathy (42). The increase in ROS production, especially O_2_·^−^, leads to excessive endogenous antioxidant capacity in the body (43). In turn, the excessive production of O_2_·^−^ leads to further production of ROS in mitochondria, creating a vicious cycle of oxidative stress (44, 45). Excessive ROS induce protein denaturation and directly cause oxidative damage to DNA (46), which is particularly serious because mitochondrial DNA is related to the electron transport chain (ETC) (47). Furthermore, metalloproteinases and other proteases are activated, causing further functional deterioration of a variety of proteins, including antioxidant enzymes (48).

## Calcium Overload and Changes in Mitochondrial Membrane Permeability

Cytoplasmic calcium homeostasis is impaired in cardiomyocytes in septic cardiomyopathy, and Ca^2+^ enters mitochondria through unidirectional transporters (49). In addition, the rapid oscillation of Ca^2+^ between mitochondria and endoplasmic reticulum also leads to mitochondrial Ca^2+^ overload, which further initiates the opening of mitochondrial permeability transition pore (mPTP) (50). The outer mitochondrial membrane is highly permeable, substances with molecular weights less than 1500 kDa can pass through it, while the inner mitochondrial membrane allows only substances with molecular weights less than 1.5 kDa to pass through it (51). Proton pumps in the inner mitochondrial membrane pump protons from mitochondrial matrix to outer chamber, forming a potential difference between inside and outside mitochondria, which is called the mitochondrial membrane potential (ΔΨm) (52). The mPTP opens intermittently in physiological state, and protons or positive ions in the outer chamber enter the inner chamber because of the potential difference, preventing the excessive accumulation of positive ions in the outer chamber (53). With Ca^2+^ overload, persistent oxidative stress, adenosine deficiency, increased phosphate concentration and mitochondrial depolarization occur, and then, the mPTP is in a mostly irreversibly opened state (54). The ΔΨM decreases rapidly, leading to ion imbalance, mitochondrial swelling and ATP depletion (55). Moreover, mPTP opening leads to the release of cytochrome c into the cytoplasm, which participates in forming apoptotic bodies with APAF-1 and the precursors of caspase-9. Apoptotic bodies activate caspase-9 facilitated by deoxy-ATP (dATP), and caspase-9 then enzymatically cleaves caspase-3 to activate it, which starts the caspase-induced apoptosis cascade of reactions that ultimately leads to cell apoptosis (56, 57). In addition, electrons produced by the mitochondrial ETC can no longer be transported to oxygen molecules, resulting in the termination of OXPHOS and the inhibition of ATP synthesis (58). To maintain the ΔΨm, mitochondria then negatively regulate F0F1 ATP synthase, leading to hydrolysis of the remaining ATP (59).

## Mitochondrial Biogenesis and Mitophagy

The levels of NO, ROS and the ratio of adenosine monophosphate (AMP)/ATP increase during septic cardiomyopathy. These changes trigger mitochondrial biogenesis (60). The main mechanism of mitochondrial biogenesis is the activation of the PGC family, especially PGC-1 α. PGC-1 α is synergistically activated, and its expression leads to the increasing expression of transcription factors, mediating the expression of nuclear proteins required for the transcription and replication of nucleus- and mitochondria-encoded OXPHOS subunits and mitochondrial DNA, transcription of OXPHOS assembly factor and mitochondrial protein import components (61). Mitochondrial biogenesis stands for the growth and division of mitochondria (62). The recovery of cardiac function in septic cardiomyopathy depends partly on mitochondrial biogenesis (63). The mechanism of mitochondrial biogenesis is debated. Some studies have shown that the clearance of damaged mitochondria in sepsis can be compensated by mitochondrial biogenesis rate, producing new mitochondria. However, other studies have shown that mitochondrial biogenesis, even as a compensatory mechanism of mitochondrial dysfunction, may lead to greater mitochondrial dysfunction by disrupting the complicated processes of gene transcription and mitochondrial dynamics. In any case, mitochondrial biogenesis in septic cardiomyopathy is insufficient to compensate for mitochondrial dysfunction (64, 65). The process opposing mitochondrial biogenesis is mitochondrial autophagy (66). Mitochondrial autophagy is a mechanism by which mitochondria eliminate dysfunctional mitochondria (67). However, it is unclear whether mitochondria clear dysfunctional mitochondria only through autophagic mechanisms and/or whether autophagy is involved in programmed cell death in septic cardiomyopathy. Recent research has not clarified the relationship between mitochondrial biogenesis and mitochondrial autophagy.

The recovery of mitochondrial function is closely related to the reversal of cardiac pump function; therefore, an increasing number of in-depth targeted intervention studies are needed to prevent or even reverse mitochondrial dysfunction. Guidelines for systematic evaluation of sepsis can improve prognosis and reduce mortality. However, there is no specific treatment for sepsis complicated with damage to some organs, including the heart. Further studies on the mechanisms of mitochondrial dysfunction in septic cardiomyopathy may supply a novel strategy to supplement the treatment options.

## Epigenetics and LncRNAs

Epigenetics is the study of stable genetic phenotype inheritance that does not intervene the gene sequence (68). Epigenetic modifications regulate many biological processes, including development and cell differentiation and proliferation (69). Currently, epigenetic mechanisms include the modification of DNA and proteins closely related to DNA. That is, epigenetic studies focus on the modification and influence of chromatin structure, mainly including chromatin remodelling (including advanced folding of chromatin and connections with the nuclear matrix), DNA methylation, histone modification and noncoding RNA-related mechanisms (70, 71). The reversibility of epigenetic regulation provides a targeted treatment strategy for epigenetically modified components and new ideas for innovative clinical treatment methods.

LncRNAs are endogenous RNAs with transcript lengths of more than 200 nucleotides, which do not possess the function of encoding protein. NcRNAs account for 98% of the human genome, and lncRNAs account for 80-90% of all ncRNAs (72, 73). LncRNAs are currently considered to be key epigenetic regulators (74). With increasing and in-depth research on whole-genome sequencing and function, the structure and function of lncRNAs have been found to be particularly complex (75). Although there is no consensus on the functional classification of lncRNAs, four main types are currently recognized: signals, decoys, guides and scaffolds (76). As signals or decoys, lncRNAs participate in the activation or inhibition of gene. As guides, they enlist chromatin-modifying enzymes to regulate gene expression in a cis/trans manner. As scaffolds, they enlist a variety of proteins to synthesize ribonucleoprotein complexes that regulate chromatin or histones (77). According to the classification of gene structure, lncRNAs are mainly divided into sense lncRNAs, antisense lncRNAs, intronic lncRNAs, long intergenic lncRNAs (or lincRNAs), enhancer RNAs (or erRNAs), and circular RNAs (or circRNAs) (78). LncRNAs interact with various molecules to form RNA-RNA, RNA-DNA and RNA-protein complexes, which play important roles in chromatin modification (79).

## LncRNAs and Cardiovascular Diseases

Mutation or abnormal expression of lncRNAs is closely relevant to cardiovascular diseases (80, 81). Published research results mainly refer to MIAT, ANRIL, LIPCAR, and Braveheart. As early as 2006, scholars explored the relationship between MIAT and myocardial infarction. MIAT single-nucleotide polymorphisms can cause changes in the expression of myocardial infarction-related proteins (82). Overexpression of ANRIL can change sites of chromosome 9p21 that are closely relevant to the pathogenesis of coronary atherosclerosis (83). Further studies showed that ANRIL expression was positively related to the severity of coronary atherosclerosis (84). It was discovered that LIPCAR expression was upregulated during the early stage of heart failure and downregulated during the late stage, and therefore, changes in LIPCAR expression can be used to predict the risk of late cardiovascular events (85). It has been confirmed that Braveheart is closely relevant to the differentiation of mouse cardiomyocytes. Studies have shown that PRC2 can inhibit the genes necessary for the differentiation and development of cardiac cells, such as the MesP1 gene, and Braveheart can interact with SUZ12 in the PRC2 complex to further control the expression of MesP1. When the expression of Braveheart is lower than normal, mouse embryonic stem cells did not differentiate into normal cardiomyocytes, which limited heart development (86).

## Effects of Regulated LncRNA Expression on Mitochondrial Function

Mitochondria are important multifunctional organelles participating in various basic biological processes (87). The integrality of mitochondrial structure and function is significant to maintain the stability of the intracellular environment. Currently, it is generally believed that the stability of the intracellular environment depends on various mitochondrial pathways regulating energy conversion and ATP production, involving ETC and tricarboxylic acid cycle (TCA) (88). Mitochondria have genetic system independent of the nucleus, and the mitochondrial genome has a complete expression mechanism (89). However, the scale of the mitochondrial genome is small (90). The biological function of mitochondria does not solely depend on the mitochondrial genome; it also depends on nucleus-encoded proteins, which are synthesized in the cytoplasm and transported into mitochondria through specific mechanisms. In other words, mitochondrial energy metabolism and intracellular environment stability depend on the simultaneously coordinated regulation and expression of the nuclear genome and mitochondrial genome (91). Increasing evidence has shown that lncRNAs can act as messengers between nucleus and mitochondria, and participate in regulating of diverse pathways (92). However, the potential regulatory mechanisms may be very complex, and relevant research is ongoing.

LncRNAs can regulate mitochondrial function and dynamics at different levels (93). Abnormal regulation of lncRNAs leads to abnormal synthesis of ATP and ROS, thus contributing to the pathological development of many diseases. Currently, research on lncRNA regulation of mitochondrial function mainly focuses on cardiovascular diseases, neurodegenerative diseases and tumour diseases (94–96). As mentioned above, cardiomyocytes are enriched with many mitochondria, and mitochondrial dysfunction is closely relevant to the pathogenesis of cardiovascular diseases.

## Effects of LncRNA Regulation on Mitochondrial Dysfunction in Septic Cardiomyopathy

As previously mentioned, various mechanisms of mitochondrial dysfunction in septic cardiomyopathy have been reported. According to the literature, recent research on lncRNAs participating in the regulation of mitochondrial dysfunction in septic cardiomyopathy has mainly focused on decreases in ATP production, mitochondrial NO production and oxidative stress. Additionally, studies have shown that lipopolysaccharide (LPS) can induce an increase in ROS, a decrease in ΔΨm, the release of cytochrome c, and the upregulation of caspase-9 and caspase-3 in the cytoplasm, ultimately leading to cardiomyocyte apoptosis (97).

Cheng Xing Peng et al. explored the regulatory role of MIAT in septic myocardial injury. They found that MIAT knockdown significantly inhibited the production of mitochondrial ROS in LPS-treated HL-1 cells. In addition, the ratio of reduced glutathione to oxidized glutathione (GSH/GSSH) decreased with increasing malondialdehyde (MDA) content. This result suggested that MIAT aggravated myocardial damage by promoting oxidative stress. It was confirmed that MIAT acted on miR-330-5p directly to upregulate the TRAF6/NF-κB pathway, promoting inflammation and oxidative stress in LPS-induced cardiomyopathy (98).

RMRP inhibits the posttranscriptional regulatory effect of miR-1-5p on HSPA4 in LPS-induced mitochondrial damage. Overexpression of RMRP can significantly inhibit the decline in ΔΨm, the level of intracellular ROS, and the expression of cytoplasmic cytochrome c, caspase-9 and caspase-3, thereby inhibiting cardiomyocyte apoptosis (99). Bin Shan et al. discussed H19 regulation in septic cardiomyopathy. H19 can reduce mitochondrial inner membrane damage by regulating mitochondrial membrane potential by regulating miR-93-5p/SORBS2 pathway, thereby inhibiting mitochondrial apoptosis. Inflammatory factors, involving TNF-α, IL-1β and IL-6, were markedly downregulated in LPS-induced cardiomyocytes overexpressing H19. The expression of cytochrome c in mitochondria was upregulated, while that in cytoplasm was downregulated. This result indicated that the overexpression of H19 alleviated inflammation and mitochondrial apoptosis in LPS-induced cardiomyocytes (100). Studies have also pointed out that knocking down SOX2OT can significantly enhance cardiac function, inhibit the decline in ΔΨm, and reduce the production of mitochondrial ROS in mice with septic cardiomyopathy, while upregulating SOX2OT can reverse all of these effects. Through further research on the regulatory mechanism, it was ultimately concluded that SOX2OT aggravated mitochondrial dysfunction by downregulating the expression of SOX2, thereby affecting the prognosis of septic cardiomyopathy (101).

Studies on the involvement of lncRNAs regulating mitochondrial energy metabolism in septic cardiomyopathy are also ongoing. Dongshi Liang et al. found that the increased expression of Xist is related to the decreased level of both PGC-1α and ATP, which suggested that inhibiting the expression of Xist enhanced the production of ATP, reducing sepsis-induced myocardial injury (102).

Although the aforementioned lncRNAs have been confirmed to participate in septic cardiomyopathy by regulating mitochondrial function and apoptosis, it is still unclear whether other lncRNAs are involved in regulating mitochondrial functions, and the specific regulatory mechanisms of participating lncRNAs are also unknown. To date, using gene chip hybridization technology, researchers at Zhejiang University identified 471 upregulated lncRNAs and 804 downregulated lncRNAs in myocardial tissues of septic mice. Ultimately, this group found that partial lncRNAs are mainly enriched in inflammation, immunity, energy metabolism and cell death, and predicted that certain lncRNAs may participate in mitochondrial dysfunction (103). All these results provide strong theoretical support for the continuing study of the involvement of lncRNAs in mitochondrial dysfunction in septic cardiomyopathy.

## Conclusion and Perspective

LncRNAs will increasingly become targets for the intervention and treatment of septic cardiomyopathy, and the mechanism to target is closely related to lncRNA involvement in mitochondrial dysfunction. Finding intervention measures to prevent and treat mitochondrial damage is significant to improved treatment and prognosis of patients with septic cardiomyopathy. Although research on biomarkers for use in assessing the severity and prognosis of septic cardiomyopathy is ongoing, no clear markers with both sufficient sensitivity and specificity have been identified to date. Recent research has found that CitH3 may be recognized as a reliable blood biomarker for diagnosis and prognosis of sepsis (104). LncRNAs may be potential biomarkers for evaluating the severity and prognosis of septic cardiomyopathy, and they will also be the focus of the next phase of our research.

## Author Contributions

WC and SL conceived the review. SL wrote the manuscript. WC revised the manuscript. All authors contributed to the article and approved the submitted version.

## Funding

This work is supported by the project of scientific-technology plan in Shenyang, China (Grant number 19-112-4-068).

## Conflict of Interest

The authors declare that the research was conducted in the absence of any commercial or financial relationships that could be construed as a potential conflict of interest.

## Publisher’s Note

All claims expressed in this article are solely those of the authors and do not necessarily represent those of their affiliated organizations, or those of the publisher, the editors and the reviewers. Any product that may be evaluated in this article, or claim that may be made by its manufacturer, is not guaranteed or endorsed by the publisher.
